# Assess and rehabilitate body representations *via* (neuro)robotics: An emergent perspective

**DOI:** 10.3389/fnbot.2022.964720

**Published:** 2022-09-08

**Authors:** Gaia Risso, Michela Bassolino

**Affiliations:** ^1^School of Health Sciences, Haute École spécialisée de Suisse occidentale (HES-SO) Valais-Wallis, Sion, Switzerland; ^2^The Sense Innovation and Research Center, Sion, Switzerland; ^3^Robotics, Brain and Cognitive Sciences (RBCS), Istituto Italiano di Tecnologia, Genoa, Italy; ^4^Laboratoire MySpace, Université de Lausanne, Lausanne, Switzerland

**Keywords:** body representations, neurorobotic, rehabilitation, stroke, amputee, haptic, eating disorder (ED), sensorimotor function impairment

## Abstract

The perceptions of our own body (e.g., size and shape) do not always coincide with its real characteristics (e.g., dimension). To track the complexity of our perception, the concept of mental representations (model) of the body has been conceived. Body representations (BRs) are stored in the brain and are maintained and updated through multiple sensory information. Despite being altered in different clinical conditions and being tightly linked with self-consciousness, which is one of the most astonishing features of the human mind, the BRs and, especially, the underlying mechanisms and functions are still unclear. In this vein, here we suggest that (neuro)robotics can make an important contribution to the study of BRs. The first section of the study highlights the potential impact of robotics devices in investigating BRs. Far to be exhaustive, we illustrate major examples of its possible exploitation to further improve the assessment of motor, haptic, and multisensory information building up the BRs. In the second section, we review the main evidence showing the contribution of neurorobotics-based (multi)sensory stimulation in reducing BRs distortions in various clinical conditions (e.g., stroke, amputees). The present study illustrates an emergent multidisciplinary perspective combining the neuroscience of BRs and (neuro)robotics to understand and modulate the perception and experience of one's own body. We suggest that (neuro)robotics can enhance the study of BRs by improving experimental rigor and introducing new experimental conditions. Furthermore, it might pave the way for the rehabilitation of altered body perceptions.

## Mental body representations

Our body mediates the interactions with the external environment, through the senses and movements. Moreover, the body is itself an object of perception, whose current status is transmitted to the brain *via* multiple bodily signals. Bodily information is conveyed by visual and external auditory cues, but also by tactile, proprioceptive, vestibular, or interoceptive signals. These multisensory and motor signals are integrated into coherent models of the body, i.e., body representations (BRs), that are stored in our brain encoding and tracking the state of the body in time and space (De Vignemont, 2016; Riva, 2018; Bassolino and Serino, 2021). Intriguingly, the way we perceive the body does not always coincide with the physical body. Studies revealed systematic distortions of certain properties of the body (e.g., position and size) across healthy individuals (Bassolino et al., 2014; Longo, 2018; Galigani et al., 2020; Sorrentino et al., 2021), and in different clinical conditions such as amputation, stroke, or neuropsychiatric disorders (Thakkar et al., 2011; Keizer et al., 2013; Blanke et al., 2016; Case et al., 2019).

Following the description of patients' altered BRs, multiple, separate body representations with specific characteristics and functions have been described (Schwoebel et al., 2002; de Vignemont, 2010). So far, no consensus has been reached on the exact number and functions of different body representations (de Vignemont, 2010; Kammers et al., 2010; Canzoneri et al., 2013). The lack of a comprehensive model has determined a widespread terminological and conceptual confusion on BRs (Gallagher, 1986; de Vignemont, 2010). Thus, precise and quantitative methods are required to isolate, control, and assess the contribution of certain sensory information (e.g., vision or proprioception) in the various BRs. The next section highlights how (neuro)robotics can provide support in investigating BRs to improve our understanding of their definitions and functions.

## (Neuro)robotics to assess body representations

In the context of BRs, a distinction between explicit and implicit BRs has been proposed (Canzoneri et al., 2013; Longo, 2015a). Explicit BRs include perceptual, conceptual, or emotional subjective knowledge that humans consciously have about their own body (Cash and Brown, 1987; de Vignemont, 2010; Longo, 2015a; Bassolino and Serino, 2021). Explicit BRs can be assessed by asking subjects directly, as through questionnaires [e.g., the Body Shape Questionnaire, Cuzzolaro et al., 2006; the Bath Complex Regional Pain Syndrome (CRPS) Body Perception Disturbance Scale, Ten Brink et al., 2021; the Affected Limb Perception Questionnaire, see https://osf.io/p6v7f], or *via* other protocols requiring participants to compare, draw, or recognize their perceived image (e.g., the image marking methods, optical distortion methods, depictive method) (Gardner and Brown, 2010; Longo et al., 2015). In contrast, there is knowledge about our bodies that we are not constantly aware of, yet we implicitly use it to move and interact with objects. For instance, when we venture into a small cave, we can cross it without hitting the rock by considering the dimensions of the cave, but also implicitly the size of our body. Various tasks have been designed to tap into implicit BRs without asking the subject directly. Some protocols focus on assessing metric features requiring participants to localize body parts or somatic sensations in the body (Longo and Haggard, 2010). Participants' judgments are used to infer the perceived implicit length/width of the body parts by calculating the distance between two reported localizations (e.g., the arm length is estimated by calculating the distance between the perceived localization of the index and the elbow). Then, the perceived and real dimensions are compared to detect eventual bias (Bassolino et al., 2014; Saulton et al., 2016; Longo, 2018). Often, these tasks benefit from motion tracking systems to precisely register the perceived and real positions of the body (Peviani and Bottini, 2018; Galigani et al., 2020), thus reducing potential human errors resulting from manual measurements. These protocols could exploit robotics to guide or assist the participants' movements enabling experimental paradigms in active conditions, which have already been showed to tap into metric BRs involved in the action (e.g., see Peviani and Bottini, 2018; Peviani et al., 2020 where participants move the tested non-visible hand to reach external visual targets). Indeed, robotic workstations can be programmed to include and control different parameters (hand positions, speed, low friction, mechanical robustness, etc.) allowing them to mimic the dynamic characteristics of natural movement (Krebs et al., 1998; Grange et al., 2001; Casadio et al., 2006). Movement-assistance robots would be particularly useful to improve the current assessment of BRs metrics in patients with sensorimotor deficits (Bassolino et al., 2022). For instance, motor impairments and spasticity (i.e., muscle stiffness, tightness, and rigidity) (Pantano et al., 1995; Sommerfeld et al., 2004) would prevent the use of active tasks in some patients with poststroke, thus limiting their assessment to static tasks (Longo and Haggard, 2012; Bassolino et al., 2015), or motor imagery skills (Shahvaroughi-Farahani et al., 2021). Assisting technology would allow exploiting residual motor functions in these patients by promoting the use of active tasks to investigate BR subserving action. In addition, robotic protocols have been largely used to manipulate the external environment, for instance by applying forces through a manipulandum as in the seminal studies by Shadmehr and Mussa-Ivaldi (1994) to study motor learning. Results showed gradual adaptation of the subjects' dynamics to the force-field manipulation by suggesting the ability to predict and compensate for the changes in the external environment (Shadmehr and Mussa-Ivaldi, 1994; Burdet et al., 2001). These adaptative protocols would be used to study plastic effects on BR due to motor learning in healthy subjects and patients (Sarlegna et al., 2010; see also next section). More generally, studying BR by considering the perspective of motor control would drive major achievements in the understanding of the origin and functions of distortions in body perception (Bassolino and Becchio, Under Review).

Other tasks used to evaluate BRs only partially exploit the potential support of robotics. The Tactile Distance Task (TDT) is a widely used experimental procedure for assessing the perception of distances between tactile stimuli (Tamè et al., 2016; Longo and Golubova, 2017; Tosi and Romano, 2020). The rationale behind TDT is that no tactile receptor provides information on the distance between two touches on the skin. Accordingly, to estimate these distances, we need to map those touches on a representation retaining the metric properties of the body part being stimulated (Longo et al., 2010, 2015; Longo and Haggard, 2011). Typically, the TDTs setup consists of sticks, or a grid applied to the skin. Since the stimulations are often applied manually, many factors are hard to control such as the force or pressure of the stimulating objects, or the stimuli position. Haptic robotic devices (i.e., devices designed to enable human–machine interaction *via* the kinesthetic and/or tactile sense) (Basdogan and Srinivasan, 2002; Biggs and Srinivasan, 2002; O'Malley and Gupta, 2008) would improve the control and replicability of the experimental procedures allowing to systematically apply the pressures with predefined force in the same location.

Haptic robotics devices can also be used in the context of multisensory integration paradigms (e.g., the Phantom force-feedback devices) (Ernst and Banks, 2002; Gepshtein and Banks, 2003), where sensory modalities (e.g., vision and touch) are independently manipulated in various conditions. These multisensory protocols have also been adapted to indirectly investigate BRs, as in the case of patients suffering from anorexia nervosa where the overestimation of stimuli's width is interpreted as an alteration of the perceived size of the stimulated body region (Serino and Haggard, 2010; Risso et al., 2020), or amputees where a multisensory optimal stimulation reduces phantom sensations (Risso and Valle, 2022; Risso et al., 2022). Multisensory stimulation has also been used to induce illusions such as the rubber hand illusion (RHI) (Botvinick and Cohen, 1998) or the full-body illusion (FBI) (Lenggenhager et al., 2007) to study self-perception and specifically body ownership (i.e., the unified and coherent experience of owning our body) and agency (i.e., the subjective experience that the self is identified as the agent of the actions). Typically, the synchronized tactile stimulation of the subject's real non-visible body, together with the view of the same stimulation on a fake body, induces the illusion that the fake body feels like the participants' one. The congruency between the visuotactile stimulation is crucial inducing the illusion (Blanke et al., 2015). However, the tactile stimulation is often given manually, thus introducing possible, not measurable, incongruencies between stimulations on the real and fake limbs and likely reducing the illusion strength. Accordingly, technological versions of the RHI/FBI have been developed employing virtual reality (Tsakiris et al., 2006; Kilteni et al., 2012; Serino et al., 2022), neurostimulations (Marasco et al., 2011; Bassolino et al., 2018), and robotics (Arata et al., 2014; Salomon et al., 2017; Huynh et al., 2019), allowing to better control and synchronize the stimuli presentation, and to propose other versions of the illusions (e.g., visuomotor stimulations). For instance, to induce the motor RHI, a robotic master–slave manipulator has been used, where the subject performs unilateral movements inducing similar movements on the rubber hand (Hara et al., 2014). A similar approach has been used to induce the feeling of presence (FoP), i.e., the strange sensation that somebody is nearby when no one is actually present. The FoP resembles the presence of hallucinations described in neurological and psychiatric patients (Critchley, 1955; Brugger et al., 1996). The protocol to induce the FoP in healthy individuals provides that while standing blindfolded, participants moved their arms and the master device in front of them. These movements were sent to the slave robot, which applied tactile stimuli in real time to the participants' backs. When the tactile feedback on the back is delivered asynchronously with respect to patients' arms movements in the front, the FoP was reported (Blanke et al., 2014). Recently, this protocol has also been applied to patients with Parkinson's disease (PD) to determine the fundamental mechanisms of hallucinatory symptoms in this pathology (Bernasconi et al., 2021). The authors identified a subgroup of patients with PD showing an increased sensitivity to conflicting sensorimotor stimulation and robot-induced hallucinations. In addition, by combining MR compatible robotics to deliver sensorimotor stimulation in healthy participants and lesion network mapping in neurological patients without PD, authors showed that the frontotemporal connectivity, associated with hallucinations in healthy participants, was disrupted in patients with PD suffering from hallucinations. Moreover, this study can also be taken as an example of the use of MR-compatible robotic devices (in this case, the master–slave robot for the sensorimotor stimulation) (Hara et al., 2014) to allow controlled and reproducible bodily-related multisensory stimulation in the scanner to study the neural correlates of body perception in healthy participants and patients (see for other studies on body perception using MR-compatible robotic devices: Ionta et al., 2011; Blanke et al., 2014; Akselrod et al., 2021).

Overall, the use of neurorobotics has proved particularly helpful to study self-body perception. Accordingly, the term “cognetics” has been proposed to indicate key technological approaches that can render and combine artificial multisensory stimuli with motor signals to investigate bodily perception and consciousness (Rognini and Blanke, 2016).

**Figure 1:**
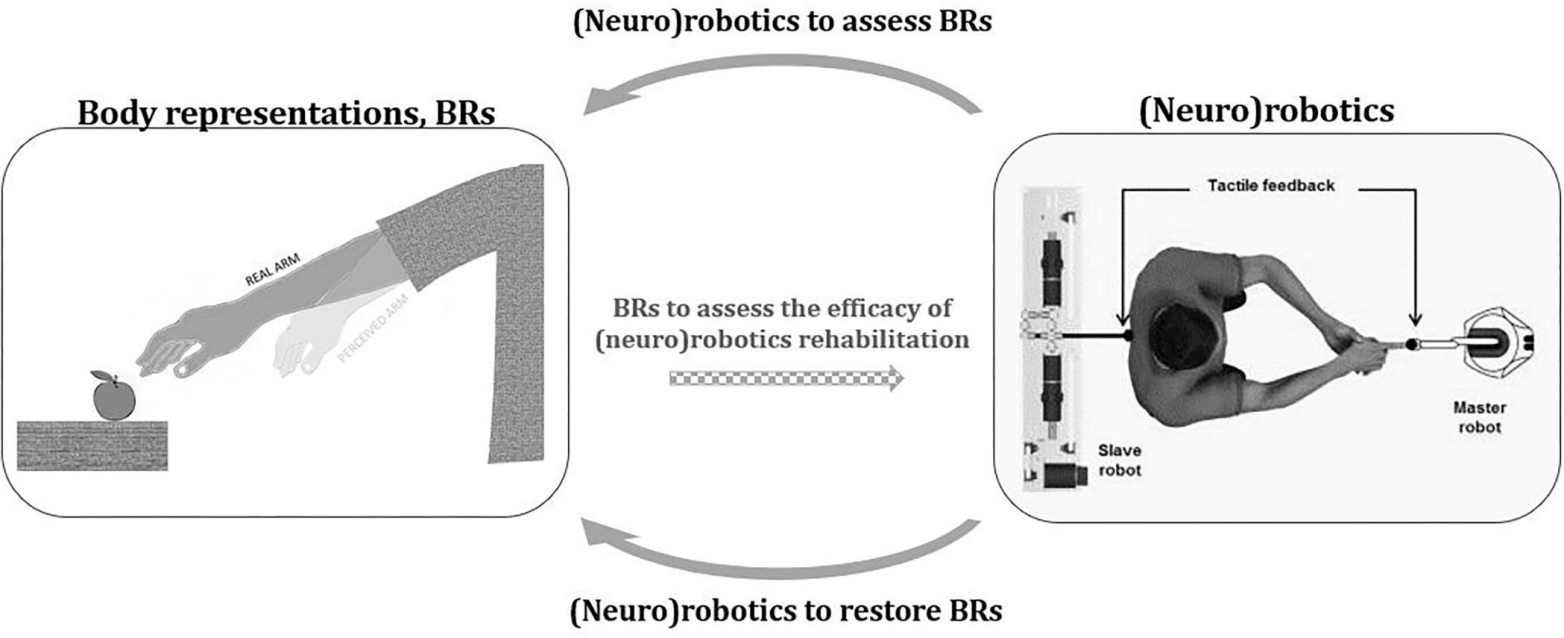
Interaction between body representations and (neuro)robotics. The figure represents the potential contribution of (neuro)robotics to the field of body representations (BR). (Neuro)robotics might enhance the study of BRs by improving experimental rigor and by allowing new empirical conditions that would be difficult to achieve without technological automation and control (top arrow). By providing controlled and precise somatosensory stimuli, also in combination with other technologies (e.g., virtual reality), (neuro)robotics may offer a significant contribution to the modulation of distorted BRs (bottom arrow). In this new perspective, the ability of robotic devices to monitor and possibly treat BRs distortions could become one of the parameters to evaluate their effectiveness (central dotted arrow). The image on the right is modified with permission from Blanke et al. (2014).

To sum up, the inclusion of robotic instruments into BRs experimental routine might support more rigorous empirical research and new experimental paradigms allowing to unveil crucial BRs' mechanisms (see upper arrow Figure 1). The costs of the robotic devices limit the applicability and development of robotics protocols evaluating BRs. Often, these technologies are not available in the research or rehabilitation centers (Wolpert and Flanagan, 2010), and require considerable expertise. Interdisciplinary collaboration among different professionals and novel affordable user-friendly and transportable device development could be an important contribution to future research.

## Distortion in mental body representations

In several pathological conditions (Keizer et al., 2013; Schott, 2014; Risso et al., 2020; Longo, 2022), abnormal perceptions of the body could be directly caused by a lack of altered inflow of sensory, proprioceptive, and/or motor information to the brain due to central or peripheral lesions (e.g., stroke or deafferentation), or because of structural damage (e.g., amputees). Alternatively, such distortions may not directly involve the somatosensory pathway, as in eating disorders (Nico et al., 2010; Keizer et al., 2012) or body integrity dysphoria (Saetta et al., 2020). Although the presence of biases in body perception can also characterize healthy subjects' perceptions (Longo and Haggard, 2011; Longo, 2017, 2022), pathologies are often accompanied by undesired feelings. For instance, amputation or deafferentation is characterized by unpleasant phantom sensations and pain (Flor et al., 2006). In patients with chronic stroke with persistent sensorimotor deficits, the affected limb can be perceived as “foreign,” “ill,” and “like dead” (Bassolino et al., 2022; Crema et al., 2022).

Noteworthy, one of the most interesting characteristics of BRs is their plasticity. BRs can reshape accordingly to sensorimotor experiences such as tool use (Canzoneri et al., 2013; Martel et al., 2016; Galigani et al., 2020), anesthesia (Paqueron, 2003), immobilization (Bassolino et al., 2015), or in case of manipulation of multisensory processing inducing illusory sensations (see the previous paragraph) (Taylor-Clarke et al., 2004; Bruno and Bertamini, 2010; Tajadura-Jiménez et al., 2012). This evidence suggests that the BRs distortions observed in patients can be restored through appropriate rehabilitative training. This is crucial given that alterations in BRs could be a detrimental factor in the functional abilities' recovery (Farne, 2004; Gialanella et al., 2005; Hammerbeck et al., 2019) and protheses' use and acceptance (Makin et al., 2017; Preatoni et al., 2021).

## (Neuro)robotics to reduce distortions in body representations

The main idea behind rehabilitative robots is to provide physical support to patients by adapting to their residual sensorimotor abilities, with the specific aim of enabling actions accomplishment and therapy support by increasing its intensity and repetitions (Casadio et al., 2009; Marini et al., 2017; Gassert and Dietz, 2018). Most rehabilitation technologies aim to restore motor impairments. Robotic technologies have also been proposed to convey tactile feedback (Dario, 1991; Reinkensmeyer and Dietz, 2016; Bicchi and Buttazzo, 2020; Handelzalts et al., 2021; Yeh et al., 2021) or reliable and quantitative proprioceptive assessments (Dukelow et al., 2010; Cappello et al., 2015). Neural interfaces based on invasive and non-invasive stimulations yielded promising results in this direction, restoring haptic sensations to individuals with somatosensory deficits (Petrini et al., 2019a,c; Risso et al., 2019; Ortiz-Catalan et al., 2020; Crema et al., 2022). Overall, these technologies allow modulating sensory modalities that are fundamental to constructing and updating BRs. However, their effects in reducing BRs distortions have been rarely assessed. Few studies exploiting neural interfaces compared the perceived body dimensions in patients with altered BRs before and after stimulation. Rognini et al. (2018) administered tactile stimulation to the phantom limb of amputees *via* intraneural implant into the residual limb nerves. Through virtual reality, such stimulation was combined with a coherent visual illumination of the patient's prosthetic hand. This visuotactile immersive experience importantly reduced telescoping phantom sensation (i.e., the phantom limb is perceived as shorter than the intact limb). Phantom limb distortions (i.e., perceiving the phantom dimensions/positions as closer to contralateral length) were also proved to decrease in a real-life environment (Graczyk et al., 2018) or when biomimetic feedback (i.e., specific encoding strategy modulating the frequency and amplitude of the stimulations to elicit more natural sensations) was delivered in a natural environment (i.e., not in virtual reality) during precision grip tasks (Valle et al., 2018). Similar results were shown in lower limb amputees, where a multisensory approach combining virtual reality and electrocutaneous non-invasive stimulation allowed to decrease the telescoping effect in one patient and the phantom leg displacement (i.e., the perceived shift in the phantom leg's position) in the second patient (Risso et al., 2022). Phantom displacement reduction was also proved in lower limb amputees provided with invasive direct nerve stimulation (Petrini et al., 2019b). Intriguingly, even if the prosthetic device usually weighs less than the limb they replace (Jones and MIT Press, 2019), amputees often perceive it as much heavier, with an important impact on prosthesis acceptance and use (Sinha et al., 2014; Handy Eone et al., 2018). Preatoni et al. (2021) showed that an amputee provided with a coherent somatosensory stimulation *via* intraneural feedback during walking reported a decrease in the perceived weight of the prosthesis (Preatoni et al., 2021).

Although studies exploiting neurorobotics-based rehabilitation to improve altered BRs mainly focus on amputees with the aim of boosting artificial limb embodiment (Makin et al., 2020), alterations in BRs have also been reported in different clinical conditions such as eating disorders, chronic pain, or stroke (Moseley et al., 2012; Keizer et al., 2013; Bassolino et al., 2022). To tackle BRs alterations, rehabilitative training exploiting multisensory stimulations has been proposed (Bolognini et al., 2015; Garbarini et al., 2015). In this sense, the potential of neurorobotics may be crucial providing controlled and precise sensory stimuli also in combination with other technologies (e.g., virtual reality) (see bottom arrow in Figure 1). Promising results in this direction are shown by a recent study assessing the effect of neurorobotics-based stimulation in patients with chronic stroke with motor deficits. After receiving a rich sensorimotor neuromuscular electrical stimulation through an array of 59 active electrodes embedded in a matrix placed on the patient's arm (helping hand), the alterations in the perceived arm dimension and the altered subjective feeling toward the affected limb were significantly reduced (Crema et al., 2022). Interestingly, alteration in BRs also involved other aspects of self-perception as body ownership, agency (Longo, 2015b), and the perception of pain (Flor et al., 2006; Makin et al., 2013, 2015; Halicka et al., 2020; Schone et al., 2022). Intriguingly, recent studies suggest that sensory stimulation *via* neurorobotics has beneficial effects on all these aspects (agency: Collinger et al., 2013; ownership: Marasco et al., 2011; and pain: Page et al., 2018; Petrini et al., 2019a). However, before any attempt at rehabilitation, our knowledge of distortions in different clinical populations should be better known (leading back to the first part of this study, higher arrow in Figure 1). For example, studies in patients with anorexia nervosa showed impairments in their implicit body experience (Keizer et al., 2013; Spitoni et al., 2015; Risso et al., 2020). A neurorobotics-based rehabilitation allowing to correct these implicit distortions could be relevant support for these patients.

In this perspective, the ability of robotic devices to monitor and possibly treat BRs distortions could become one of the parameters to evaluate their effectiveness (dotted central arrow in Figure 1). Thanks to interdisciplinary collaboration and knowledge, this change in perspective might create new techniques to improve the recovery of patients with altered BRs and sensorimotor functions and increase our knowledge on self-body perception.

## Conclusion

In conclusion, mental body representations (BRs) are a complex and multifaceted concept involved in different clinical conditions (e.g., stroke, psychiatric disease, deafferentation, amputation, etc.) whose recovery might significantly improve the patient's outcomes. The past decades have seen rapid developments in rehabilitative (neuro)robotics, which has been only recently applied to the field of BRs. The few seminal evidence on the use of neurorobotics for the evaluation and treatment of BRs described in this review encourages the further development of such an approach in patients with stroke and amputees and its extension to other pathologies (e.g., eating disorders, integrity dysphoria, deafferentation, etc.). However, given that our knowledge of BRs is still partial and growing, the application of neurorobotics for BRs rehabilitation must be accompanied by basic scientific investigation. Robotics might also be an important support in this regard, allowing more sophisticated and controlled experimental paradigms to assess unisensory and multisensory features underlying BRs.

## Data availability statement

The original contributions presented in the study are included in the article/supplementary material, further inquiries can be directed to the corresponding author/s.

## Author contributions

GR and MB co-wrote this review and made the figure. All authors authorized submission of the manuscript.

## Funding

This work was supported by the Swiss National Science Foundation (SNSF) Ambizione Grant to MB (PZ00P1_161210).

## Conflict of interest

The authors declare that the research was conducted in the absence of any commercial or financial relationships that could be construed as a potential conflict of interest.

## Publisher's note

All claims expressed in this article are solely those of the authors and do not necessarily represent those of their affiliated organizations, or those of the publisher, the editors and the reviewers. Any product that may be evaluated in this article, or claim that may be made by its manufacturer, is not guaranteed or endorsed by the publisher.
